# Loss to follow-up and HIV incidence in female sex workers in Kaiyuan, Yunnan Province China: a nine year longitudinal study

**DOI:** 10.1186/s12879-016-1854-y

**Published:** 2016-09-29

**Authors:** Yingying Su, Guowei Ding, Kathleen H. Reilly, Jessie L. Norris, Huixin Liu, Zheng Li, Guixiang Wang, Ganggang Fang, Ning Wang

**Affiliations:** 1 National Institute of Diagnostics and Vaccine Development in Infectious Diseases, State Key Laboratory of Molecular Vaccinology and Molecular Diagnostics, School of Public Health, Xiamen University, Xiamen, China; 2 National Center for AIDS/STD Control and Prevention, Chinese Center for Disease Control and Prevention, Beijing, China; 3 Kaiyuan Center for Disease Control and Prevention, Kaiyuan, China

**Keywords:** Female sex workers, HIV, Longitudinal study, Incidence, Loss to follow-up

## Abstract

**Background:**

Longitudinal studies of female sex workers (FSWs) are vulnerable to loss to follow-up (LTFU) due to this population’s high mobility and low willingness to self-identify as FSWs. LTFU in cohort studies is a critical problem and may lead to bias in estimation of incidence and exposure-outcome associations. The aim of this study was to analyze LTFU and HIV incidence and their associated factors in a 9-year longitudinal study of FSWs in Kaiyuan, Yunnan Province, China.

**Methods:**

This analysis includes all HIV-1 seronegative FSWs who were recruited into a prospective study in Kaiyuan with at least one follow-up visit after enrollment from March 2006 to November 2013. Participants were visited in 6-month intervals after enrollment. Their demographic and behavioral data and blood specimens for HIV and sexual transmitted disease testing were collected at enrollment and at each follow-up visit. The administrative censoring date was December 31, 2014. Participants were considered LTFU if their last visit occurred 1 year or more before the administrative censoring date. Univariate and multivariable Cox regression models with time-independent variables were used to investigate the hazard ratios (HR) and 95 % confidence intervals (CI) of the factors associated with LTFU and HIV acquisition.

**Results:**

Of the 1158 FSWs, 950 were defined as LTFUs (LTFU rate: 29.69, 95 % CI: 27.85–31.62 per 100 person years [PYs]), and 33 experienced HIV seroconversions (cumulative incidence: 1.06, 95 % CI: 0.74–1.47 per 100 PYs). After adjustment, we found that FSWs who used drugs were less likely to be LTFU compared with non-drug users (adjust hazard ratio [AHR]= 0.62, 95 % CI: 0.51–0.76), though FSWs who used drug were associated with a higher risk of HIV acquisition (AHR = 3.06, 95 % CI: 1.49–6.30). Also, FSWs who always used condoms with clients in the previous month were associated with a higher risk of LTFU (AHR = 1.51, 95 % CI: 1.15–1.97), while they were negative associated with new HIV infection (AHR = 0.28, 95 % CI: 0.12–0.61).

**Conclusions:**

A high LTFU rate exists in the Kaiyuan FSW cohort study, and LTFU did not occur at random. Participants retained in the cohort tended to be at higher risk of HIV acquisition, which may result in an overestimate of the incidence of HIV infection from the Kaiyuan FSW cohort.

## Background

The HIV epidemic in China remains concentrated in high risk populations rather than in the general population [1]. Since 2007, heterosexual transmission has been the primary mode of HIV transmission in China [2]. Female sex workers (FSWs) play an important role in transmitting HIV in the general population [3]. Yunnan province is a persistent hotspot for the HIV epidemic in China [4], with the highest cumulative number of HIV infection cases in China [5]. The HIV prevalence among FSWs ranged from 1.95 to 2.22 % [6] in Yunnan, which was significantly higher than national average (0.2 to 0.4 %) between 2008 and 2010, according to sentinel surveillances [7]. Kaiyuan City was defined as a hotspot of the HIV epidemic in Yunnan province [8, 9]. The estimated size of the FSW population is around 1000 to 1600 in Kaiyuan, accounting for approximately 0.7–1.2 % of all females in this city [10]. More than half of FSWs in Kaiyuan are from other cities and were more mobile than those from Kaiyuan [11].

A cohort study is the gold standard method for HIV incidence estimation and is ideal for assessing the burden of HIV among FSWs [12]. However, loss to follow-up (LTFU) in cohort studies is a critical problem and may lead to bias in estimation of incidence and exposure-outcome associations [13]. It is essential in these studies to maximize the follow-up rate as much as possible [14]. However, when conducting a cohort study, LTFU is unavoidable and the rate of LTFU typically increases over time [15]. In addition, longitudinal studies of FSWs are vulnerable to LTFU due to this population’s high mobility and low willingness to self-identify as FSWs [16, 17], especially in China where commercial sex is illegal [18].

In this analysis, we estimated the incidence of LTFU and HIV infection, and identified the factors associated with LTFU and HIV seroconversion to assess the impact of LTFU on incidence assessment in a longitudinal study of FSWs in Kaiyuan City, Yunnan Province, China.

## Methods

### Study population

The Kaiyuan longitudinal study of female sex workers (FSWs) is an open cohort study initiated in March 2006 to explore the incidence and risk factors for infection with HIV and other sexually transmitted diseases (STDs) in Yunnan, China. Details of the study have been described elsewhere [19, 20]. Briefly, outreach workers of this study recruited FSWs from all local sex work venues. Women were recruited into the study if they were at least 16 years of age, self-reported engaging in commercial sex in the last 3 months, and provided written informed consent. Participants were followed in every 6-month intervals after enrollment. Demographic and behavioral data, and blood specimens for HIV, herpes simplex virus type-2 (HSV-2), and syphilis testing were collected at enrollment and each follow-up visit. A digital fingerprint of each participant was obtained to identify participants at each visit. Efforts were made to encourage study follow-up. All staff members were well trained to build rapport with participants. Before each follow-up survey, staff contacted all study participants using previously provided contact information to remind them to attend the upcoming survey. A Red Ribbon Home, which provides health and social services to study participants, was established by local CDC staff members in 2006. The availability of these services by the local CDC also improved the compliance of participants to the study.

This analysis included all HIV-1 seronegative FSWs who had been recruited into the Kaiyuan prospective study with at least one follow-up visit after enrollment from March 2006 to November 2013. The administrative censoring date was December 31, 2014.

### Data collection

At all study visits, data on demographics and sexual and drug use behaviors were obtained by self-report from each participant through face-to-face interviews with trained local staff members. All participants received pre-test counseling and 50 Yuan (7 USD) in compensation at each visit, and HIV post-test counseling at 4–6 weeks after each visit. Participants with positive HIV or STD results were referred to local hospitals to receive the appropriate treatment.

The HIV antibody was screened by two enzyme-linked immunosorbent assay methods (ELISA-1 and ELISA-2, BioMerieux, Holland), and if positive for at least one method, confirmatory testing was conducted using Western Blot (WB, MP Biomedicals, Singapore). The HSV-2 antibody (HerpeSelect-2 ELISA IgG, Focus, Hackensack NJ) and syphilis antibody (rapid plasma reagin test, Kehua, China) were also tested. Positive rapid plasma reagin tests were confirmed by Treponema Pallidum Particle Agglutination assay (TPPA, Livzon, China).

### Statistical analysis

A new HIV-1 infection was considered when a participant’s HIV serostatus changed from negative to positive, and the date of HIV-1 acquisition was assumed to be the mid-point between the dates of the last negative test and the first positive test. Participants were considered LTFU if their last visit occurred 1 year or more before the administrative censoring date. This definition of LTFU is consistent with that used in another study by Tuboi et al. [21].

We used the following covariates in analysis: year of enrollment (2006–2007, 2008–2009, 2010–2011, 2012–2013), age (≤25 years, >25 years), marital status (married/cohabiting, unmarried/divorced/widowed), education (≤9 years, >9 years), duration of sex work (≤3 year, >3 years), drug use (no, yes), alcohol use (no, yes), number of monthly clients (≤30, >30), condom use in previous month (never or sometimes, always), charge for sex (“low”: ≤100 Yuan, “high”: >100 Yuan), HSV-2 status (negative, positive), and syphilis status (negative, positive).

Since FSWs with new HIV infections were also encouraged to remain in the study after infection, we calculated the follow-up time separately for LTFU and HIV acquisition. For the HIV incidence calculation, the follow-up time for each participant was calculated as the time between the enrollment visit and the date of HIV-1 acquisition or the last visit if the HIV status was still negative. The HIV incidence rate was defined as the number of HIV seroconversions divided by total number of person-years (PYs) of observation. In analyzing the rate of LTFU, the follow-up time for each participant was calculated as the time between the enrollment visit and the last visit. The incidence rate of LTFU was defined as the number of LTFUs divided by the total number of PYs of observation. The 95 % confidence interval (CI) for each rate was calculated using a Poisson distribution. A Cochran-Armitage trend test was used to study the underlying trends for HIV incidence and LTFU rates over time. A Kaplan Meier survival curve was used to describe the probability of LTFU. Univariate and multivariable Cox regression models with time-independent variables were used to investigate the hazard ratios (HR) and 95 % CIs of factors associated with LTFU and HIV acquisition. Variables found to be statistically significant in univariate analyses were included in the multivariable analysis to explore the independent factors for LTFU and HIV acquisition and to assess the adjusted hazard ratios (AHR) and 95 % CIs of the independent factors. All statistical analyses were conducted using SAS version 9.3 (SAS Institute, Cary, NC).

## Results

A total of 1158 FSWs with at least one follow-up visit after enrollment were included in this analysis. The mean age of FSWs was 26.7 years, with a standard deviation (SD) of 8.0 years. Most FSWs (84.9 %) were enrolled in the first 4 years (2006–2009). Only 13.8 % of FSWs reported attending school for more than 9 years. Only 27.8 % of FSWs were from Kaiyuan City; others were from elsewhere in Yunnan or from other provinces. The prices charged for sex were considered “low” (≤100 yuan) for the majority of FSWs (62.5 %). And 186 (16.1 %) FSWs self-reported at enrollment that they had a history of drug use. The respective prevalence of HSV-2 and syphilis among FSWs at enrollment was 59.0 and 7.4 % (Table 1).

**Table 1:** LTFU rate and HIV incidence among FSWs stratified by demographic and behavioral characteristics at enrollment

Variables at enrollment	*N*	%	LTFU			HIV acquisition		
			*n*	PYs	rate (95 % CI) per 100 PYs	*n*	PYs	rate (95 % CI) per 100 PYs
ALL	1158	100.0	950	3199.8	29.69(27.85–31.62)	33	3105.7	1.06 (0.74–1.47)
Year of enrollment								
2006–2007	649	56.1	555	2084.6	26.62 (24.48–28.91)	23	2015.9	1.14 (0.74–1.68)
2008–2009	334	28.8	271	844.8	32.08 (28.43–36.07)	6	825.2	0.73 (0.30–1.50)
2010–2011	92	7.9	76	177.6	42.77 (33.94–53.22)	4	171.8	2.33 (0.78–5.54)
2012–2013	83	7.2	48	92.8	51.72 (38.59–67.96)	0	92.8	0 (0–2.66)
Race								
Han	808	69.8	661	2259.6	29.25 (27.09–31.55)	22	2200	1.00 (0.64–1.49)
Minority	350	30.2	289	940.2	30.74 (27.34–34.44)	11	905.7	1.21 (0.64–2.10)
Age								
≤ 25 years	594	51.3	531	1408.6	37.70 (34.59–41.01)	14	1372.4	1.02 (0.58–1.67)
> 25 years	564	48.7	419	1791.2	23.39 (21.23–25.71)	19	1733.3	1.10 (0.68–1.67)
Marital status								
Married/cohabiting	454	39.2	358	1271.4	28.55 (25.73–31.6)	12	1239.7	0.97 (0.53–1.64)
Unmarried/divorced/widowed	704	60.8	592	1928.4	30.44 (28.05–32.98)	21	1866	1.13 (0.72–1.69)
Education								
≤ 9 years	998	86.2	820	2749.6	29.82 (27.83–31.92)	27	2667.3	1.37 (0.68–1.45)
> 9 years	160	13.8	130	450.2	28.87 (24.23–34.17)	6	438.4	1.10 (0.57–2.82)
Census registration								
Local	322	27.8	232	1031.0	22.50 (19.74–25.54)	11	1001.9	1.1 (0.58–1.9)
Other city	836	72.2	718	2168.8	33.10 (30.75–35.59)	22	2103.8	1.04 (0.67–1.55)
Charge								
Low	724	62.5	578	2127.4	27.17 (25.02–29.45)	26	2051.9	1.27 (0.85–1.83)
High	424	36.6	363	1048.4	34.62 (31.2–38.32)	7	1029.8	0.68 (0.3–1.33)
missing	10	0.9	9	24.0	-	0	23.8	-
Duration of sex work								
≤ 3 years	701	60.5	628	1571.7	39.96 (36.92–43.17)	12	1540.2	0.78 (0.42–1.32)
> 3 years	451	38.9	316	1624.8	19.45 (17.39–21.68)	21	1562.1	1.34 (0.86–2.02)
missing	6	0.5	6	3.3	-	0	3.4	
Number of clients per month								
≤ 30	1002	86.5	829	2718.8	30.49 (28.47–32.62)	23	2666.5	0.81 (0.44–1.38)
> 30	143	12.3	110	446.8	24.62 (20.33–29.55)	10	404.9	2.51 (1.29–4.45)
missing	13	1.1	11	34.2	-	0		-
Condom use								
Never/Sometimes	142	12.3	102	503.6	20.25 (16.6–24.48)	11	458.6	2.40 (1.27–4.15)
Always	1016	87.7	848	2696.2	31.45 (29.39–33.62)	22	2647.1	0.83 (0.54–1.24)
Drug use								
No	972	83.9	828	2528.4	32.71 (30.54–35)	20	2461.6	0.81 (0.51–1.23)
Yes	186	16.1	122	671.4	18.17 (15.16–21.61)	13	643.5	2.02 (1.13–3.36)
Alcohol use								
No	400	34.5	314	1109.3	28.32 (25.32–31.58)	14	1061.2	1.32 (0.76–2.15)
Yes	758	65.5	635	2090.5	30.37 (28.08–32.81)	19	2044	0.93 (0.58–1.42)
HSV-2 status								
Negative	475	41.0	411	1140.5	36.04 (32.68–39.65)	6	1124.1	0.53 (0.22–1.1)
Positive	683	59.0	539	2059.3	26.17 (24.03–28.45)	27	1981.6	1.36 (0.92–1.95)
Syphilis status								
Negative	1072	92.6	886	2931.1	30.23 (28.29–32.27)	27	2869.1	0.94 (0.63–1.35)
Positive	86	7.4	64	268.7	23.82 (18.5–30.21)	6	236.6	2.54 (1.05–5.23)

*Abbreviations*: *LTFU* loss to follow-up, *PYs* person years, *HSV-2* herpes simplex virus type-2

### Outcomes

**Fig. 1:**
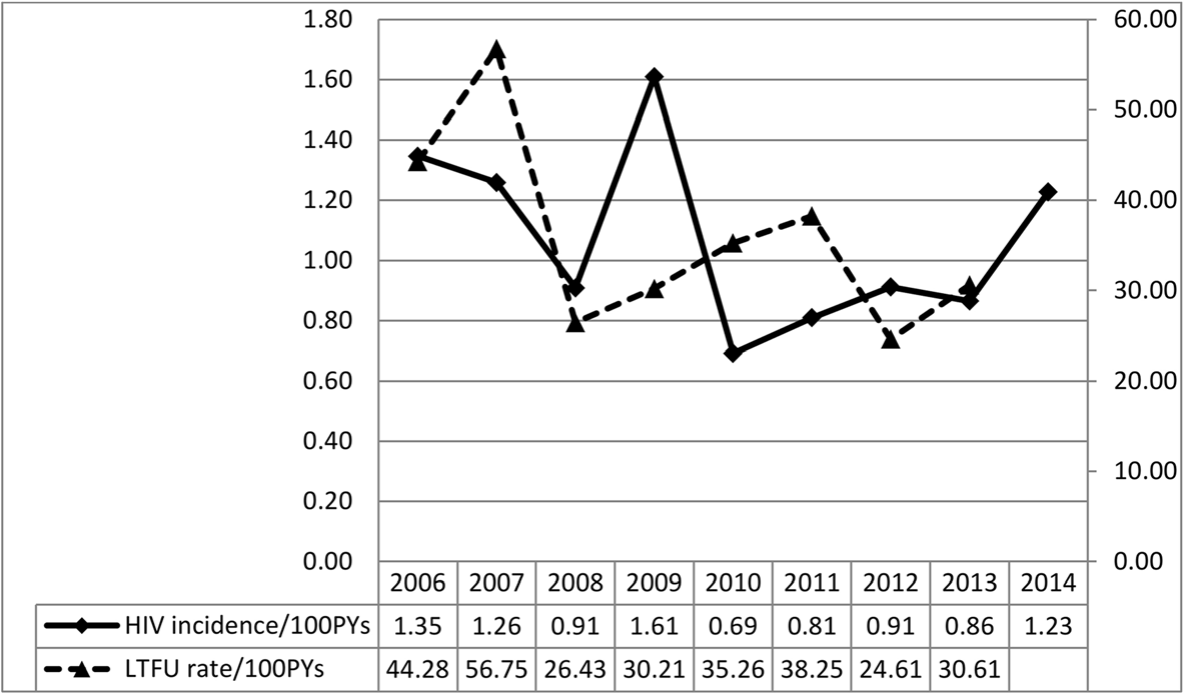
Trends in incidence of LTFU and new HIV infection

Of the 1158 FSWs, 950 (82.0 %) were considered LTFU, and 33 (2.8 %) HIV seroconverted. The mean duration of follow-up was 2.8 years (SD 2.4 years). In analyzing the rate of LTFU, 1158 FSWs contributed a total observation period of 3199.8 person years, yielding an overall LTFU rate of 29.69 (95 % CI: 27.85–31.62) per 100 PYs. As shown in Fig. 1, a downward trend was observed in the LTFU rate, which decreased from 44.28 per 100 PYs in 2006 to 30.61 per 100 PYs in 2013 (Z = -5.4, *p* < 0.001). For calculating HIV cumulative incidence, 33 new infections over 3105.7 PYs at risk yielded an incidence of 1.06 (95 % CI: 0.74–1.47) per 100 PYs. The HIV incidence rate fluctuated over time, and no statistically significant trend was observed (Z = -0.4, *p* = 0.68). We also calculated the rate of LTFU and new HIV infection stratified by demographic and behavioral characteristics at enrollment (Table 1).

### Factors associated with loss to follow-up

Factors for LTFU in univariate and multivariable Cox regression models with time-dependent variables are presented in Table 2. After adjusting for confounders, FSWs who had a history of more than 3 years’ duration of sex work at enrollment (AHR = 0.67, 95 % CI: 0.57–0.79), who were older than 25 years of age (AHR = 0.83, 95 % CI:0.71–0.96), who used drugs (AHR = 0.62, 95 % CI: 0.51–0.76)) and whose HSV-2 status was positive (AHR = 0.87, 95 % CI: 0.75–1.00) were associated with a lower risk of LTFU, while those who were single/divorced/widowed (AHR = 1.21, 95 % CI: 1.06–1.38), whose census registration was in another city (AHR = 1.32, 95 % CI: 1.13–1.54), or who always used condoms with clients in the previous month (AHR = 1.51, 95 % CI: 1.15–1.97) were associated with a higher risk of LTFU.

**Table 2:** Factors associated with loss to follow-up in the Kaiyuan study

Variables	Univariate analysis		Multivariable analysis	
	HR (95 % CI)	P	AHR (95 % CI)	P
Year of enrollment				
2006–2007	1.00	-	1.00	
2008–2009	1.12 (0.96–1.29)	0.14	1.19 (1.03–1.39)	0.02
2010–2011	1.35 (1.06–1.72)	0.02	1.35 (1.04–1.74)	0.02
2012–2013	1.66 (1.23–2.34)	0.00	2.35 (1.73–3.20)	<0.0001
Race				
Han	1.00	-		
Minority	1.06 (0.92–1.22)	0.48		
Age at enrollment				
≤ 25 years	1.00	-	1.00	-
> 25 years	0.65 (0.57–0.74)	<0.0001	0.83 (0.71–0.96)	0.01
Marital status^a^				
Married/cohabiting	1.00	-	1.00	-
Unmarried/divorced/widowed	1.23 (1.08–1.39)	0.001	1.21 (1.06–1.38)	0.007
Education				
≤ 9 years	1.00	-		
≥ 10 years	0.95 (0.79–1.15)	0.58		
Census registration				
Local	1.00	-	1.00	-
Other city	1.52 (1.31–1.75)	<0.0001	1.32 (1.13–1.54)	0.0004
Charge^a^				
Low	1.00	-		
High	1.15 (1.01–1.31)	0.03		
Duration of sex work at enrollment				
≤ 3 year	1.00	-	1.00	-
> 3 years	0.52 (0.45–0.60)	<0.0001	0.67 (0.57–0.79)	<0.0001
Number of clients per month^a^				
≤ 30	1.00	-		
> 30	0.97 (0.78–1.19)	0.76		
Condom use^a^				
Never/Sometimes	1.00	-	1.00	-
Always	1.56 (1.20–2.03)	0.0009	1.51 (1.15–1.97)	0.003
Drug use^a^				
No	1.00	-	1.00	-
Yes	0.51 (0.42–0.61)	<0.0001	0.62 (0.51–0.76)	<0.0001
Alcohol use^a^				
No	1.00	-		
Yes	1.26 (1.10–1.45)	0.001		
HSV-2 status^a^				
Negative	1.00	-	1.00	-
Positive	0.74 (0.64–0.86)	<0.0001	0.87 (0.75–1.00)	0.05
Syphilis status^a^				
Negative	1.00	-		
Positive	0.78 (0.60–1.03)	0.08		

*Abbreviations*: *HSV-2* herpes simplex virus type-2, *HR* hazard ratio, *AHR* adjusted hazard ratio

^a^Time-dependent variables

### Risk factors for new HIV infection

The univariate and multivariable risk factors associated with new HIV infection are shown in Table 3. In multivariable analysis, FSWs who always used condoms with clients in the previous month (AHR = 0.28, 95 % CI: 0.12–0.61) and those whose charges for sex were classed as “high” (AHR = 0.30, 95 % CI = 0.10–0.86) had a significantly lower risk of becoming infected with HIV, while FSWs who used drugs were more likely to acquire HIV (AHR = 3.06, 95 % CI: 1.49–6.30).

**Table 3:** Risk factors associated with HIV acquisition in the Kaiyuan study

Variables	Univariate analysis		Multivariable analysis	
	HR (95 % CI)	P	AHR (95 % CI)	P
Year of enrollment				
2006–2007	1.00			
2008–2009	0.66 (0.27–1.65)	0.37		
2010–2011	2.11 (0.71–6.32)	0.18		
2012–2013	-			
Race				
Han	1.00			
Minority	1.37 (0.66–2.82)	0.39		
Age at enrollment				
≤ 25 years	1.00			
> 25 years	1.05 (0.52–2.12)	0.88		
Marital status^a^				
Married/cohabiting	1.00			
Unmarried/divorced/widowed	0.86 (0.43–1.73)	0.67		
Education				
≤ 9 years	1.00			
≥ 10 years	1.68 (0.73–3.87)	0.22		
Census registration				
Local	1.00			
Other city	0.95 (0.47–1.95)	0.89		
Charge^a^				
Low	1.00		1.00	
High	0.21 (0.07–0.59)	0.003	0.30 (0.10–0.86)	0.02
Duration of sex work at enrollment				
≤ 3 year	1.00			
> 3 years	1.76 (0.85–3.62)	0.12		
Number of clients per month^a^				
≤ 30	1.00			
> 30	2.45 (1.06–5.70)	0.04		
Condom use^a^				
Never/Sometimes	1.00		1.00	
Always	0.26 (0.12–0.55)	0.0005	0.28 (0.12–0.61)	0.001
Drug use^a^				
No	1.00		1.00	
Yes	3.49 (1.75–6.98)	0.0004	3.06 (1.49–6.30)	0.002
Alcohol use^a^				
No	1.00			
Yes	0.20 (0.20–0.80)	0.01		
HSV-2 status^a^				
Negative	1.00			
Positive	3.66 (1.11–12.06)	0.03		
Syphilis status^a^				
Negative	1.00			
Positive	3.23 (1.39–7.49)	0.006		

*Abbreviations*: *HR*, hazard ratio, *AHR* adjusted hazard ratio, *HSV-2* herpes simplex virus type-2

^a^Time-dependent variables

## Discussion

To our knowledge, this is the first study to analyze LTFU incidence and assess the impact of LTFU on incidence assessment in a longitudinal study of FSWs in China. The LTFU rate was 29.69 (95 % CI: 27.85–31.62) per 100 PYs and the incidence of HIV infection was 1.06 (95 % CI: 0.74–1.47) per 100 PYs in this longitudinal study in Kaiyuan City, Yunnan province. In a longitudinal study of Kenyan FSWs [22], the LTFU rate was 23.4 per 100 PYs, which is similar to our study, while the incidence of HIV acquisition was 4.5 per 100 PYs, which is significantly higher than our results.

In a longitudinal study to analyze incidence and risk factors for HIV infection, it is important to maintain high retention of participants to avoid bias in estimates of incidence and exposure-outcome associations [14]. However, FSWs are considered difficult to enroll and follow up [17]. It is common in cohort studies of FSWs to find that more than 30 % FSWs are lost to follow-up [23, 24]. Therefore, it is quite important to estimate bias from LTFU in prospective cohort studies of HIV acquisition in FSWs. In analyzing the risk of LTFU, we found that LTFU in our cohort did not occur at random [25]. FSWs who were single/divorced/widowed, whose census registration was in another city and who always used condoms with clients in the previous month were associated with a higher likelihood of LTFU. We assume that single/divorced/widowed FSWs and those who came from other cities had greater flexibility to move to other cities to engage in sex work. Also, some single FSWs might get married and no longer work as FSWs. On the other hand, FSWs who had a history of sex work of more than 3 years at enrollment, who were older than 25 years of age, who were drug users and whose HSV-2 status was positive were more likely to be retained in the cohort. This might be explained by the lower mobility rate among FSWs whose duration of sex work was longer than 3 years or who had a history of drug use [11]. Also most FSWs who used drugs received methadone treatment and other services from local CDC staff, which may have improved their compliance with our study. In our study, participants with an STD were referred to local hospitals where they could receive a 60 % discount on treatment [19]. This may have also improved compliance among those who were diagnosed with HSV-2.

Though various factors were associated with LTFU, among these only condom use and drug use were associated with new HIV infection. We found that FSWs who used drugs were at higher risk of HIV acquisition than those who did not, which was also shown in another study in Vietnam [26]. We also found that FSWs who always used condoms with clients in the previous month were negatively associated with new HIV infection, which has also been recognized in many other studies [27–29]. The above factors indicate that FSWs who were LTFU in the Kaiyuan cohort had a lower risk of acquiring HIV. A similar finding was also demonstrated in the Kenyan study mentioned above [22].

Another important issue in cohort studies of FSW is that the duration of sex work among females was shorter compared with other populations at high risk for HIV infection [30], which increased the difficulty of follow up. Fazito et al.’s study documented that the mean duration for sex work in Asia was 2.94 years, which was much lower than in other regions of the world [30]. A study by sexuality researcher S.M. Pan demonstrated that the mean duration of sex work in China was around 2 years and that most FSWs transferred to a non-sex work job after this time [31]. The mean duration of follow-up was 2.8 years in our study, which is close to the expected mean duration of sex work. It is possible, therefore, that most FSWs who were LTFU in our study were no longer engaged in sex work and were at lower risk of HIV acquisition.

This study has several limitations. First, we explored only the association between LTFU or HIV acquisition and internal factors in FSWs, such as demographic and behavioral factors. External aspects such as social policy and economic conditions were not considered in this study. Since 2010, several government crackdowns on sex work have been implemented in China, which increases the mobility of sex workers [18]. Also, Kaiyuan City has experienced a shrinkage in sexual markets following the closing of many large factories. This could also force FSWs to migrate to other cities for work. Second, details on the reasons for dropout were not collected in this study, since once FSWs did not return to the study, it was quite difficult for us to contact them. Third, behavioral factors were self-reported by FSWs, which may undermine the validity of the results in our study. Finally, we did not observe many incident cases of HIV, which may have impacted the statistical power to conduct multivariable analysis.

## Conclusions

In conclusion, there was a high LTFU in this cohort of Kaiyuan FSWs and LTFU did not occur at random. We found that participants retained in our cohort tended to be at higher risk of acquiring HIV, which may result in overestimating the incidence of HIV infection among Kaiyuan FSWs. Detailed reasons for LTFU require further study, and external factors such as social policy and social economy also need to be considered in the future.

## Abbreviations

AHR: Adjusted hazard ratio
CI: Confidence interval
FSWs: Female sex workers
HR: Hazard ratio
HSV-2: Herpes simplex virus type-2
LTFU: Loss to follow-up
PYs: Person years
